# Editorial: Updates on memory modulation in health and disease

**DOI:** 10.3389/fnbeh.2023.1205371

**Published:** 2023-05-04

**Authors:** Magdalena Miranda, Marcelo Giachero, Noelia V. Weisstaub, Juan Facundo Morici

**Affiliations:** ^1^Institute of Functional Genomics, University of Montpellier, CNRS, Inserm, Montpellier, France; ^2^Laboratorio de Memoria y Cognición Molecular, Instituto de Neurociencia Cognitiva y Traslacional, Consejo Nacional de Investigaciones Científicas y Técnicas-Fundación INECO-Universidad Favaloro, Buenos Aires, Argentina; ^3^Institut du Fer a Moulin, UMR-S 1270, INSERM and Sorbonne Univerité, Paris, France

**Keywords:** memory, neuromodulation, neurodegeneration, neurotransmission dysfunction, memory impairment

One of the most intriguing questions in the memory field is how memory systems can be modulated in their nature and strength. By sculpting the contribution of different neuronal populations and target structures in the brain, neurotransmitters and neuromodulators are key players of high-order cognitive functions. So, which factors can modulate the memory process? The main objective of this issue is to approach this multifactorial question from a multidisciplinary perspective. Articles in this issue highlight general properties that make neuromodulatory systems crucial players in the behavioral neuroscience field and explain how different neuropathological conditions can alter these systems.

The long-term stabilization of information in the brain requires the reorganization of pre-existing networks. Hippocampal interneurons play a pivotal role in this matter, by controlling the size of the neural ensemble encoding new memories (Stefanelli et al., 2016). Indeed, it has been shown that inhibition of parvalbumin (Karunakaran et al., 2016; Xia et al., 2017), and somatostatin (Adler et al., 2019; Morales et al., 2021) interneurons in the hippocampus alters encoding of contextual memories. It is thought that neuromodulation shapes memory strength by configuring microcircuits and target structures that are recruited by an encoded event. Critically, hippocampal interneurons highly express acetylcholine (ACh) receptors (Morales et al., 2008; Son and Winzer-Serhan, 2008), suggesting a role of this neurotransmission system in the modulation of the hippocampal inhibitory activity. However, how Ach signaling mediates memory formation by modulating the excitatory/inhibitory balance in the hippocampus remains unclear. In this Issue, Goral et al. have provided evidence suggesting that the loss of GABA co-transmission from Ach-activatable interneurons alters spatial and contextual fear memories.

Spatial memory deficits are one of the most common cognitive symptoms in neurodegenerative disorders that selectively affect the medial-temporal lobe (MTL), such as Alzheimer's disease (AD) (Visser et al., 2002; Berron et al., 2020). Interestingly, AD disease leads to a bulk of neuromodulatory changes that impact over several neurotransmitter systems (Fahnestock et al., 2002; Rissman et al., 2007; Dinamarca et al., 2012; Revett et al., 2013; Kandimalla and Reddy, 2017; Wang et al., 2019; Chen et al., 2022). The hippocampal formation (HPC), one of the most affected MTL structures in AD, presents neurons tuned to fire at particular places in the environment (i.e., *place cells)* that are crucial for encoding spatial information (O'Keefe and Dostrovsky, 1971). Decoding accuracy and stability of hippocampal spatial representations is modulated by Ach (Sun et al., 2021) and NMDAR activity (Tonegawa et al., 1996; Cabral et al., 2014). Remapping, the process by which spatial hippocampal information stored in *place cells* is modified (Muller and Kubie, 1987; O'Keefe and Burgess, 1996; Leutgeb et al., 2005; Colgin et al., 2010), is thought to be an essential mechanism by which hippocampal formation maintains updated internal representations of changing environments. Hippocampal alteration in AD might lead to remapping impairments and potentially to the typical symptomatology of this disease. In this regard, the article by Silva and Martinez reviewed evidence pointing out the mapping and remapping disruption in the HPC as a possible circuit mechanism involved in deficits observed in AD. Moreover, they discuss how tau mediated changes in NMDA and AMPA receptors function in hippocampus-entorhinal cortex (HP-EC) region could contribute to these deficits.

Memories are susceptible to near-learning experiences that can change the internal state of an individual and influence memory strength (Moncada et al., 2011; Tyng et al., 2017; Tarder-Stoll et al., 2020). Neuromodulators are key players for experience-dependent changes in memory strength. For example, exercise and environmental enrichment, are interventions that are known to increase several neuromodulatory systems such as BDNF levels and lead to an increase in memory performance (Grech et al., 2018; Xu et al., 2021). Brain-Derived Neurotrophic Factor (BDNF) has been implicated in the formation and stabilization of the synapses (Cohen-Cory et al., 2010), and postulated as a marker of the occurrence/progression of many mnemonic symptoms that are common to different neuropathological conditions (Miranda et al., 2019). In addition, BDNF is also a key neuromodulator of the nociceptive response (Thompson et al., 1999; Pezet et al., 2002). This evidence suggests possible connections between nociceptive and memory systems. Indeed, a recent study elucidates the interaction between the concentration of this neurotrophic factor and tDCS-dependent alleviation of cognitive impairment observed in fibromyalgia (Dos Santos et al., 2018), a disease characterized by chronic neuropathic pain (Wood, 2007). In this issue, Caumo et al. describe a positive relationship between levels of BDNF and severity of cognitive impairment in subjects that responded to the conditioned pain modulation test, which is not seen in non-responders. These results open the possibility of the involvement of BDNF in moderating the effect of chronic pain on cognitive functions.

One of the most important questions in the study of memory is how individuals learn to avoid real or perceived dangers. Memories resulting from these experiences are of clinical interest as maladaptive memories are thought to be at the core of anxiety-related disorders observed in humans (Gazarini et al., 2023). To tackle this challenge, several animal models that mimic aspects of human aversive memories have been developed over the years. In this regard, fear conditioning has been the most widely used procedure to study the processing of emotional memories in rodents (LeDoux, 2000). However, due to the great complexity of rodent models, it is vitally important to study these processes using other animal models. Here, Pribadi and Chalasani review studies in invertebrates like *Aplysia californica, Drosophila melanogaster*, and *Caenorhabditis elegans* showing mechanisms underlying learning and memory processes conserved across these species. This review pays particular attention to predator-induced fear in these three organisms opening potential applications to more naturalistic trials.

In sum, this issue addresses the role of neuromodulatory transmission in shaping microcircuit memory encoding and explains how pathological conditions can impact memory function influencing several neurotransmitter systems, and crucially, how different animal models can help to understand these processes. Also, it discusses the development of naturalistic invertebrate animal models for studying learning and memory processes in maladaptive-memory formation.

## Author contributions

JM and MM contributed substantially to the concept and design of the article, as well as making the original draft of the article. MG and NW revised it critically for important intellectual content. All authors contributed to the article and approved the submitted version.
